# New cell separation technique for the isolation and analysis of cells from biological mixtures in forensic caseworks

**DOI:** 10.3325/cmj.2011.52.293

**Published:** 2011-06

**Authors:** Cai-xia Li, Gui-qiang Wang, Wan-shui Li, Jiang-ping Huang, An-quan Ji, Lan Hu

**Affiliations:** 1 Institute of Forensic Science, Ministry of Public Security, Beijing, China; 2 Key Laboratory of Forensic Genetics, Ministry of Public Security, Beijing, China; 3 Chinese Peoples Public Security University, Beijing, China

## Abstract

**Aim:**

To isolate mucosal cells of the perpetrator in a sexual assault case from a complex mixture of his mucosal cells and the victim’s skin by micromanipulation prior to genomic analysis.

**Methods:**

To capture and analyze mucosal cells we used the micromanipulation with on-chip low volume polymerase chain reaction (LV-PCR). Consensus DNA profiles were generated from 5 replicate experiments.

**Results and conclusions:**

We validated the use of micromanipulation with on-chip LV-PCR for genomic analysis of complex biological mixtures in a fatal rape case. The perpetrator’s mucosal cells were captured from nipple swabs of the victim, and a single-source DNA profile was generated from cell mixtures. These data suggest that micromanipulation with on-chip LV-PCR is an effective forensic tool for the analysis of specific cells from complex samples.

In sexual assault cases, swabs from the victim’s vagina and skin surface are collected and analyzed for DNA and forensic evidence. In cases where no sperm cells are detected, alternative sources of DNA must be identified. The Forensic Science Service has developed a method to isolate male cells from vaginal swabs in azoospermic sexual assault cases (1,2). Further, skin surface swabs from sites such as the face and nipple can provide saliva samples from the assailant. Kenna et al (3) found that salivary DNA persists on skin for a minimum of 96 hours, providing a sufficient window to collect and process samples. Swabbing a large area of the victim’s skin surface, however, can yield a mixed profile of cells from both the victim and perpetrator. Unfortunately, such a mixed profile of cells can often be of limited use.

We combined micromanipulation with on-chip low volume polymerase chain reaction (LV-PCR) to identify and isolate individual cells (4). Micromanipulation was performed based upon the distinct cellular morphology of mucosal cells (of perpetrator origin) compared with epithelium of the victim. The micromanipulation method is more economical than other techniques yielding similar precision, such as laser capture microdissection (LCM). An individual genotype was obtained that was in concordance with the genotype of the suspect.

The main aim of this study was to explore the utility of micromanipulation and LV-PCR for genotyping an individual from a complex biological mixture.

## Case background

In September 2009, a female cadaver was found partially clothed and with a wooden stick inserted in her vagina. Autopsy indicated that the victim died from massive hemorrhage of the pelvic and abdominal cavity, caused by the wooden stick. During interrogation, the apprehended suspect claimed to have never seen the victim, and denied having any sexual contact with her.

There were no semen stains or related biological evidence detected on the victim. The wooden stick was stained with a substantial amount of the victim’s blood, but no DNA or fingerprints were detected belonging to the perpetrator. Right nipple swabs were the only biological evidence that contained a sample of the perpetrator’s DNA, but the swabbing yielded a mixed profile of male/female sample and DNA processing did not yield usable results. Re-analysis of the nipple swabs by micromanipulation cell separation method was employed to genotype the perpetrator’s DNA for forensic analysis.

## Material and methods

### Routine DNA detection from swab samples

Following swabbing, a sample of the cotton was treated with MagAttract^®^ DNA Mini M48 kit (Qiagen, Hilden, Germany) to extract DNA according to the manufacturer’s guidelines. The equivalent of 1 ng DNA was amplified using the AmpFiSTR Identifiler^®^ kit (Applied Biosystems, Foster City, CA, USA) according to the manufacturer’s specifications. Positive control (AmpFiSTR^®^ Control DNA 9947A, Applied Biosystems, 0.1 ng/µL) and negative control (no DNA template) DNA amplifications were performed.

One microliter of amplified DNA was denatured in 10 µL of loading buffer, which was composed of HI-DI^TM^ formamide (Applied Biosystems, Warrington, UK) and LIZ^TM^-500 size standard mixture (Applied Biosystems, Warrington) in a proportion of 500:1 (volume in volume). Electrophoresis was performed on a 3130 XL Genetic Analyzer (Applied Biosystems, Warrington) using a 10-second injection time, followed by data analysis using Genemapper ID V3.2.1 software (Applied Biosystems, Warrington).

### Cell separation and detection method

*Step 1 – Sample preparation.* A sample of the cotton swab was incubated in TNE buffer (10mM Tris-HCL, pH 8.0; 10mM NaCl; 0.1 mM EDTA) at 37°C for 20 minutes. After centrifugation at 9000 × g for 3 minutes and removal of the supernatant, the cell pellet was re-suspended in 30 µL of TNE buffer and pipetted onto a microscope slide.

*Step 2 – Cell capture and transfer.* Micromanipulation was performed under an inverted microscope (Olympus, Tokyo, Japan) with Transfer Man NK2 micromanipulator (Eppendorf, Hamburg, Germany). Sterile glass capillaries (Eppendorf) with an inner diameter of 80 µm were employed to transfer cells. The AG480F AmpliGrid^®^slide (Advalytix AG, Munich, Germany) was used as a collection platform for cell deposition and on-chip LV-PCR. Five replicates of the experiment were conducted for each sample, consisting of 3 cells each.

*Step 3 – Cell lysis and on-chip LV-PCR.* For cell lysis, 0.75 µL of Proteinase K (0.4 mg/mL) was added to each reaction position and covered with 5 µL of mineral oil (Advalytix AG). Samples were incubated at 56°C for 40 minutes and boiled for additional 10 minutes. Thermal cycling was performed using an AmpliSpeed Cycler (Advalytix AG). The PCR mixture contained 4.2 µL of PCR Reaction Mix, 2.2 µL of Primer Mix, and 1 unit of AmpliTaq Gold DNA Polymerase from the AmpFiSTR Identifiler^®^ kit (Applied Biosystems). An aliquot of the mixture (0.75 µL) was added to each reaction position after cell lysis. Two positive controls (AmpFiSTR^®^ Control DNA 9947A, 0.1 ng/µL) were applied to every slide. Two negative controls (no DNA template) were performed on each slide using the same cell lysis buffer and PCR reaction reagents. PCR conditions were as follows: 95°C for 11 minutes; 28 cycles of 94°C for 20 seconds, 59°C for 1.25 minutes, and 72°C for 1.25 minutes, followed by 60°C for 45 minutes.

*Step 4 – Electrophoresis and analysis.* PCR products (total of 1.5 µL) were transferred to 10 µL of loading buffer. Electrophoresis was performed exactly as described earlier. Consensus DNA profiles were generated from the alleles that were triplicated in 5 replicate PCR reactions (5,6). The relative fluorescence unit detection threshold was set at 50. A heterozygote pair was called if 3 of the 5 amplifications showed a peak balance greater than or equal to 50%. An allele had to appear in all 5 amplifications to be considered a homozygote. The presence of an additional allele in 2 of the 5 amplifications was considered to indicate allelic dropout and the locus was marked with a “Z.”

## Results

**Figure 1:**
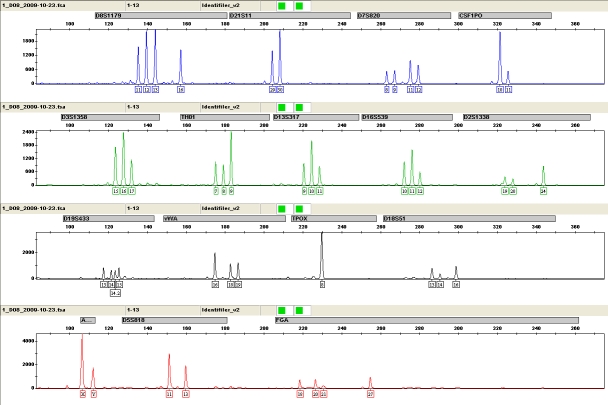
Mixed DNA profile of a perpetrator in a sexual assault case obtained by the routine method.

**Figure 2:**
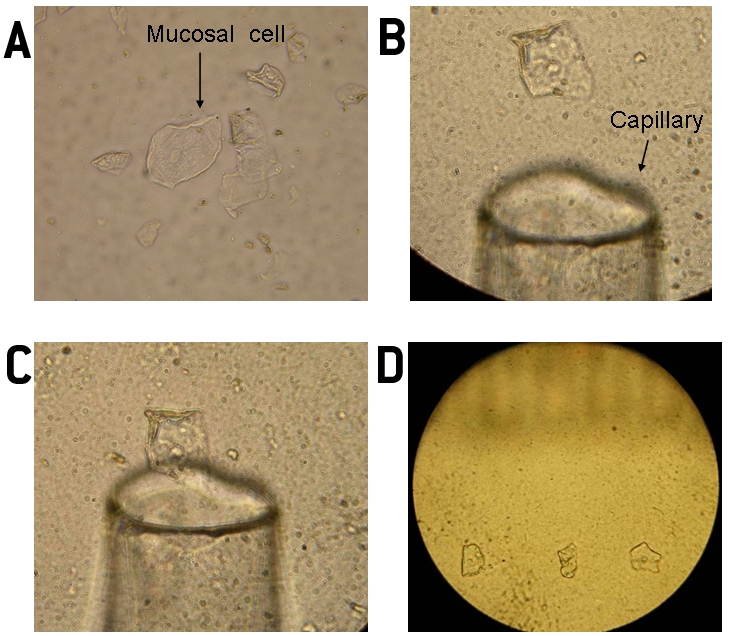
Mucosa cell isolation and collection by micromanipulation. (**A**) Identification of cells with intact nuclei; (**B**) moving capillary to cells; (**C**) capture of cells with a micro capillary; and (**D**) transfer of cells to a low-volume polymerase chain reaction slide.

PCR analysis of the mixed cell population was performed by the routine method, and a mixed DNA profile was obtained (Figure 1), which included STR alleles of both the victim and the suspect. Alternatively, male mucosal cells were isolated by micromanipulation from a swab of the victim’s nipple and DNA genotypes were obtained. Sterile glass capillary were employed to transfer the cells to the reaction spot of the AmpliGrid^®^ slide (Figure 2). Three cells with intact nuclei were captured and analyzed, and 5 replicate reactions were conducted.

**Figure 3:**
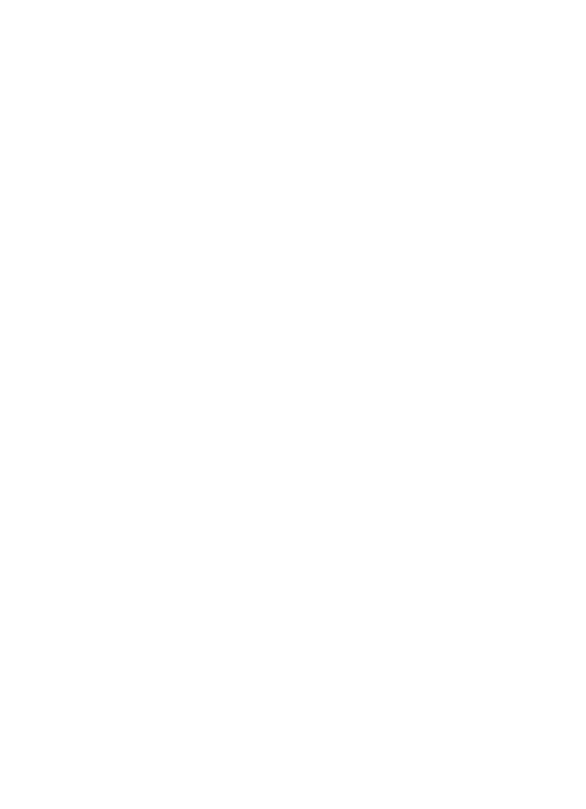
Electropherograms of each of the 5 replicates (**A-E**) performed by micromanipulation and low volume polymerase chain reaction. Red circles indicate allele dropout and arrows indicate allele drop-in.

Five electropherograms were obtained by replicated experiments performed by micromanipulation and LV-PCR (Figure 3A-E). Detailed genotyping results were summarized and a consensus DNA profile was generated (Table 1). These results are concordant with the DNA profile of the suspect, providing important evidence for this casework.

**Table 1:** Detailed genotyping results of 5 replicated experiments performed by micromanipulation and low volume polymerase chain reaction.

	Replicates					
Short tandem repeat locus	A	B	C	D	E	Consensus profile
D8S1179	11, 16	11, 16	11, 16	11, 16	11, 16	11, 16
D21S11	30, 30	30, 30	30, 30	30, 30	30, 30	30, 30
D7S820	8, 9	8, 9	8, 9	8, 9	8, 9	8, 9
CSF1PO	10, 11	10, 11	10, 11	10, 11	10, 11	10, 11
D3S1358	16, 17	16, 17	16, 17	16, 17	16, 17	16, 17
TH01	7, 8	7, 8	7, 7	7, 8	7, 8	7, 8
D13S317	9, 11	9, 11	9, 11	9, 11	9, 11	9, 11
D16S539	11, 12	11, 12	11, 12	11, 12	11, 12	11, 12
D2S1338	19, 20	19, 20	19, 20	20, 20	19, 20	19, 20
D19S433	12.2, 14, 14.2	14, 14.2	14, 14.2	14, 14.2	14, 14.2	14, 14.2
vWA	16, 16	16, 16	16, 16	16, 16	16, 16	16, 16
TPOX	8, 8	8, 8	8, 8	8, 8	8, 8	8, 8
D18S51	14, 16	14,16	14,16	14, 16	14,16	14, 16
Amelogenin	X, Y	X, Y	X, Y	X, Y	X, Y	X, Y
D5S818	13, 13	13, 13	13, 13	13, 13	13, 13	13, 13
FGA	18, 27	18, 27	18, 27	18, 27	18, 27	18, 27

## Discussion

Allelic drop-in (Figure 3A) and allelic drop-outs (Figure 3C and 3D) can be observed in electropherograms obtained by the cell separation method. Previous reports also addressed such phenomena in low template DNA (5,6). To overcome this issue, 5 replicate analyses were performed, in which 3 cells were collected for each sample. Composite DNA profiles were generated from alleles that were observed at least in triplicate in 5 replicate PCR reactions.

A skin swab taken during a rape investigation may contain, in addition to skin cells from the victim, cells derived from the perpetrator. Isolation of cells, such as mucosal cells, from a sample including skin cells can be performed based upon cell morphology and size. For instance, skin surface cells usually have no nucleus, while mucosa cells have an intact nucleus. Further, oral epithelial cells are larger than nipple skin surface cells (mean length ± standard deviation, 107.67 ± 15.30 µm vs 57.17 ± 10.96 µm, respectively, as measured by ocular micrometer, detailed results are shown in web extra material))(web extra material 1) . Together, these discrepancies allow for simple separation by micromanipulation under a microscope.

Cell folding and deformity on the cotton swab results in difficulty in differentiating cells by either size or morphology. We selected only the cells with an intact nucleus and obtained a DNA profile of a male perpetrator that is consistent with the suspect. A report by Schulz et al (7) identified a new technique that can be used in addition to the techniques described in this report to discriminate between skin and mucosal cells, using cytoskeleton analysis. As our method utilized only morphological analysis, incorporation of clearly defined tests to identify cell type would increase the reliability of micromanipulation. We expect that a combined approach would yield more precise results.

In addition to the analysis of swabs from victim’s skin surface, micromanipulation and low template PCR can also be applied to other biological mixtures. An example is the isolation of vaginal cells from penis swabs of sexual assailants (8). Sperm cell isolation can also be carried out in this platform, but it is time-consuming and labor-intensive owing to the small size of spermatozoa.

The micromanipulation platform is composed of two parts: an inverted microscope and a micromanipulator. Using this platform, the sample preparation process is simple. The capture processes begins shortly after a cell suspension is pipetted to a glass slide, with no need for smearing and drying a membrane slide as required by the LCM technique. Thus, the micromanipulation platform is cost-effective and easily established in many forensic laboratories. In fact, the total cost of this platform is approximately half of the cost for a LCM system. The primary drawback of micromanipulation compared to LCM, however, is that the cell capture process is less automated (9-11), and extensive training is necessary to become an experienced manipulator.

In addition to performing sufficient replication experiments, great caution should be taken in laboratory practice and internal validation of micromanipulation and LV-PCR to ensure reliability of the method. Caragine et al have provided explicit guidelines for forensic genetics laboratories dealing with low-template DNA (12). As such, to prevent laboratory based contamination, we have established a BSL-2 bio-safety laboratory for cell separation and detection experiments. All consumables and water were treated to remove DNA. Negative controls were also taken to check for possible contamination.

In conclusion, the method described in this report could be a very useful tool in criminal workcases to identify specific cells in complex biological samples. Micromanipulation with LV-PCR is an efficient and affordable alternative for forensic DNA analyses.

## References

[1] Murray C, McAlister C, Elliott K (2007). Identification and isolation of male cells using fluorescence in situ hybridisation and laser microdissection, for use in the investigation of sexual assault.. Forensic Sci Int Genet.

[2] McAlister C (2011). The use of fluorescence in situ hybridisation and laser microdissection to identify and isolate male cells in an azoospermic sexual assault case.. Forensic Sci Int Genet.

[3] Kenna J, Smyth M, McKenna L, Dockery C, McDermott SD (2011). The recovery and persistence of salivary DNA on human skin.. J Forensic Sci.

[4] Li CX, Qi B, Ji AQ, Xu XL, Hu L (2009). The combination of single cell micromanipulation with LV-PCR system and its application in forensic science.. Forensic Sci Int Genet.

[5] Gill P, Whitaker J, Flaxman C, Brown N, Buckleton J (2000). An investigation of the rigor of interpretation rules for STRs derived from less than 100 pg of DNA.. Forensic Sci Int.

[6] Budowle B, Eisenberg AJ, van Daal A (2009). Validity of low copy number typing and applications to forensic science.. Croat Med J.

[7] Schulz MM, Buschner MG, Leidig R, Wehner HD, Fritz P, Häbig K (2010). A new approach to the investigation of sexual offenses-cytoskeleton analysis reveals the origin of cells found on forensic swabs.. J Forensic Sci.

[8] Drobnic K (2003). Analysis of DNA evidence recovered from epithelial cells in penile swabs.. Croat Med J.

[9] Elliott K, Hill DS, Lambert C, Burroughes TR, Gill P (2003). Use of laser microdissection greatly improves the recovery of DNA from sperm on microscope slides.. Forensic Sci Int.

[10] Sanders CT, Sanchez N, Ballantyne J, Peterson DA (2006). Laser microdissection separation of pure spermatozoa from epithelial cells for short tandem repeat analysis.. J Forensic Sci.

[11] Vandewoestyne M, Van Hoofstat D, Van Nieuwerburgh F, Deforce D (2009). Automatic detection of spermatozoa for laser capture microdissection.. Int J Legal Med.

[12] Caragine T, Mikulasovich R, Tamariz J, Bajda E, Sebestyen J, Baum H (2009). Validation of testing and interpretation protocols for low template DNA samples using AmpFlSTR Identifiler.. Croat Med J.

