# Supplementary material for: New cell separation technique for the isolation and analysis of cells from biological mixtures in forensic caseworks
Source: Croat Med J. 2011 Jun;52(3):293–8. doi: 10.3325/cmj.2011.52.293 (PMC3118714; doi:10.3325/cmj.2011.52.293)
Supplement: Supplementary Table 1 [file CroatMedJ_52_s001.pdf]

Oral epithelial cells

NO.	Length (μm)
1	150
2	130
3	105
4	90
5	120
6	110
7	100
8	100
9	115
10	120
11	90
12	80
13	100
14	110
15	110
16	120
17	80
18	110
19	100
20	90
21	110
22	110
23	110
24	130
25	100
26	120
27	90
28	110
29	120
30	100
Average	107.67
SD	15.30

Nipple skin surface cells

NO.	Length (μm)
1	80
2	60
3	50
4	50
5	50
6	50
7	50
8	50
9	50
10	90
11	60
12	50
13	45
14	80
15	70
16	50
17	50
18	60
19	50
20	60
21	50
22	40
23	60
24	60
25	60
26	60
27	50
28	60
29	60
30	60
Average	57.17
SD	10.96
